# Lessons learned from a randomized controlled trial on a home delivered meal service in advanced cancer patients undergoing chemotherapy: a pilot study

**DOI:** 10.1186/s40795-021-00407-5

**Published:** 2021-02-16

**Authors:** Vera IJmker-Hemink, Nora Lize, Sandra Beijer, Natasja Raijmakers, Geert Wanten, Manon van den Berg

**Affiliations:** 1 Department of Gastroenterology and Hepatology - Dietetics and Intestinal Failure, Radboudumc, Nijmegen, The Netherlands; 2 Netherlands Comprehensive Cancer Organisation, Utrecht, The Netherlands; 3 Netherlands Association for Palliative Care (PZNL), Utrecht, the Netherlands; 4 Department of Gastroenterology and Hepatology, Radboudumc, Nijmegen, The Netherlands

**Keywords:** Pilot study, Cancer, Nutritional intervention, Chemotherapy, Qualitative interviews

## Abstract

**Background:**

Performing a randomized controlled trial (RCT) in the field of nutrition is challenging and success highly depends on understanding the factors that influence recruitment and dropout of participants. Our aim was to assess the feasibility of a RCT that evaluated a home delivered meal service in advanced cancer patients while receiving chemotherapy.

**Methods:**

This pilot RCT aimed to enroll 20 participants who were randomized into the home delivered meal service group or usual care group. Study procedures took place before chemotherapy (T0), 3 weeks after T0 (T1), 6 weeks after T0 (T2) and 3 months after T2 (T3). All information regarding recruitment, dropout and study procedures was recorded. Patient satisfaction was assessed by in-depth interviews.

**Results:**

Over 7 months, 20 of 41 approached patients (49%) were included, followed by a dropout rate of 35%. At baseline, hand grip strength (*n* = 8/16), the Short Physical Performance Battery (*n* = 12/16) and nutritional intake (n = 8/16) had the highest rate of missing values. Study procedures were not experienced as burdensome and planning of these procedures in line with fixed hospital appointments contributed to this low burden. Keeping the symptom diary was mentioned as being burdensome.

**Conclusions:**

It is feasible to conduct a RCT on a home delivered meal service in advanced cancer patients during chemotherapy, although recruitment is challenging. Close contact of patients with recruiting personnel is essential to sustain motivation. To increase compliance with the study protocol it is important to carefully instruct participants on how to complete questionnaires and to emphasize to use these in the communication with their practitioners.

**Trial registration:**

ClinicalTrials.gov NCT03382171.

**Supplementary Information:**

The online version contains supplementary material available at 10.1186/s40795-021-00407-5.

## Background

Cancer patients who receive treatment such as chemotherapy experience a variety of nutrition-related symptoms such as loss of appetite, nausea and taste changes. These nutrition-related symptoms interfere with the patient’s ability to eat and to enjoy meals, leading to impaired nutritional intake, deterioration of the nutritional status and decreased quality of life [1, 2]. Several clinical studies suggest that patient satisfaction with regard to quality of meals promotes nutritional intake during hospitalization [3, 4]. Adapting meals in line with nutrition-related symptoms might be a successful strategy to improve patient satisfaction and nutritional intake. Studies show a lack of care for nutritional problems experienced by cancer patients, leading to worsening of nutrition-related symptoms, hospitalization and a poorer prognosis [5–11]. On the other hand, adequate nutritional care and high quality food provision support the patient’s nutritional status and quality of life [3, 8].

Research efforts on nutritional care so far mainly focused on the effects of optimizing hospital meal services to improve nutritional intake. However, this feature is becoming increasingly critical for patients at home in light of the ongoing shortening of hospital stay [12]. Moreover, although most cancer patients receive their chemotherapy cycles in the hospital, they mostly recover in between these periods at home. Extending hospital nutritional care to the home setting, including meal services, is therefore becoming increasingly relevant to improve nutritional intake and possibly overall cancer care outcomes, including the prevention of hospital readmissions [13–15].

Due to the multiplicity of oncological diseases and treatment related nutritional symptoms there is a need for studies on effective nutritional interventions especially in the home situation. However, RCTs in this field are challenging due to specific barriers such as delayed recruitment, patient burden and the logistical process specifically for nutritional interventions in the home setting [16, 17]. For example, recruitment could be delayed because of low compliance with the nutritional intervention or a higher motivation to participate and comply in drug trials [17]. Hence, the success of any RCT highly depends on appreciating the factors that influence recruitment and loss of participants during follow up in general and also to gain insight in nutrition related challenges. The identification of such barriers and practical challenges but also facilitators is important when designing future trials. This pilot therefore assessed the feasibility of a RCT to evaluate a home delivered meal service in advanced cancer patients undergoing chemotherapy, as compared to usual care. We assessed recruitment, reasons for dropout, patient burden of the study protocol and satisfaction and give recommendations for future interventions in this setting.

## Methods

### Study design and population

This pilot study was performed at the Radboudumc in Nijmegen, the Netherlands prior to the start of a prospective RCT on the effect of a home delivered meal service on quality of life (ClinicalTrials.gov NCT03382171). The Medical Ethics Committee of the Radboudumc indicated that no formal approval was required for this study (2016–3043). The aim of this pilot study was to enroll 20 participants and investigate our feasibility objectives. Inclusion criteria for eligibility were Dutch-speaking adults aged 18 years or older, who had been diagnosed with metastatic colorectal or gynecological malignancies starting with 3-weekly scheduled chemotherapy. In addition, patients needed to live within a 40 km radius from the cities Nijmegen or Veghel and used exclusively oral nutrition. Exclusion criteria included renal insufficiency (MDRD-GFR < 60 ml/min and/or proteinuria), food allergies or a planned vacation during the intervention period. The inclusion criteria were expanded because of a low inclusion rate in the first 3 months as described in the results section. This expansion meant that instead of only recruiting patients with advanced colorectal or gynecological malignancies receiving a 3-weekly chemotherapy schedule, also curatively treated cancer patients receiving at least a 2-weekly chemotherapy schedule were eligible. Exclusion criteria included renal insufficiency (MDRD-GFR < 60 ml/min and/or proteinuria), food allergies, swallowing or passage problems as in head and neck cancer patients or a planned vacation during the intervention period.

### Nutritional intervention

The intervention (home delivered meal service) implied the use of six protein-rich dishes per day and was based on the FoodforCare (FfC) meal service that is currently in use in the Radboudumc [18]. Participants allocated to the intervention group received a morning shake, two lunch meals, snack, dinner and dessert for each day (average energy 1553 kcal/day, average protein 60.8 g/day) during 3 weeks. These dishes were prepared, packed for refrigerator storage and delivered to the participants two times per week. The menu consisted of a 4-week rotating seasonal menu and all participants received the same menu. In addition, participants received an information leaflet on their personal protein requirements (1.2 g/kg body weight), protein content of the dishes and a so-called, self-made, protein-measure which they could use to register their own protein intake to gain insight in whether their daily requirements were met. Since breakfast and drinks were not included in the intervention, advices about protein-rich choices were added to the leaflet including the protein content of these products. Breakfast was not included because the freshness of bread, for example, could not be guaranteed considering the dishes were provided two times per week. Participants allocated to the control group received usual care and sustained their own usual diet. These participants did not receive any nutritional advice except when they were seen by a dietitian as part of usual care. In both groups, dietitians were notified about the study participation in case individual dietary counselling was required. To minimize attitude modification the following measures were included: counselling started before randomization, dietitians are expected to follow the standard hospital protocols for nutritional counselling and dietitians were instructed to refer participants to the research team when they received questions about their participation.

### Study procedures

**Fig. 1:**
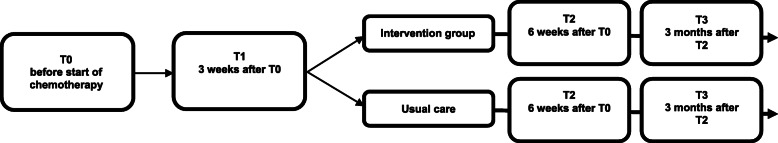
Study design of the pilot study

The study was introduced to eligible patients by a clinical nurse specialist (CNS) before the start of chemotherapy and those who were interested received an information leaflet. Also, patients were asked for consent to be contacted by the coordinating researcher to discuss the study in more detail. All participants gave written informed consent before entering the study. Patients who agreed to participate were randomized into the intervention group (home delivered meal service) or the usual care group with stratification for tumor type and the emetogenicity of the prescribed chemotherapy regimen [19]. Randomization was performed using the electronic data capture system Castor EDC by the coordinating researcher (VIJ). Given the nature of the intervention it was not possible to blind participants and/or investigators. Study procedures were performed at four fixed time points during the study period i.e. before the first cycle of chemotherapy (T0), 3 weeks after T0 at the second cycle of chemotherapy (T1), 6 weeks after T0 at the third cycle of chemotherapy (T2) and 3 months after T2 (T3) (Fig. 1). The intervention period started at T1 until T2 and lasted 3 weeks. All procedures were performed by a trained nutritionist/dietitian at the outpatient clinic or at the participants’ home.

### Primary outcome

The primary outcome of this pilot study was the feasibility of conducting a RCT concerning a home delivered meal service in advanced cancer patients undergoing chemotherapy, as compared to usual care. Feasibility was assessed by the following aspects:

- Recruitment: evaluated by the number of patients who were approached, and those who were included or excluded. All relevant information behind eligibility and participation was recorded by the coordinating researcher (VIJ).
- Dropout rates: recorded by the coordinating researcher, including reasons for dropouts.
- The feasibility of study procedures: assessed by recording data availability and reasons behind missing data.
- Patient satisfaction: assessed by in-depth interviews.

The following study procedures were performed at all time points:

- Quality of life of the participants was assessed by the validated EORTC-QLQ C30, a 30-item instrument [20].
- Caregiver quality of life was assessed by the validated Caregiver Reaction Assessment (CRA) which consists of 24 questions including both negative and positive aspects of caregiving experience [21].
- Nutritional intake was measured by a two-day food diary filled in by participants on 1 week day and 1 weekend day before each measurement and cross-checked by a dietitian [22].
- Nutritional status was determined by the validated Patient Generated- Subjective Global Assessment (PG-SGA) and by measuring body weight on a calibrated weighting scale (Seca 877) [23, 24].
- Hand grip strength was measured using a hand-held dynamometer (Lafayette Instrument Company) and is a simple, non-invasive evaluation of muscle strength [25, 26].
- The physical performance of the participants was assessed using the Short Physical Performance Battery (SPPB) and the Karnofsky Performance Status (KPS) [27, 28]. The SPPB comprises three components: balance, gait speed and chair-stand time [27]. The KPS was assigned by the treating physician [28].

Furthermore, medication use was self-reported and the severity of symptoms was graded in the ‘Utrecht Symptom Diary’ every day for 3 weeks during the intervention period (between T1 and T2) [29, 30].

Participants were asked to participate in an interview about satisfaction with regard to participation in the study and the study procedures. These interviews were conducted by one researcher according to the topic list provided in Additional file A [31]. Participants were also asked to grade the level of the overall burden of their participation between 0 (low burden) and 10 (high burden). The topic list was developed in collaboration with an expert group comprising dietitians, and nutritional and qualitative researchers. Interviews were conducted at a place of choice, mostly with the participant alone (*n* = 9) and sometimes together with their informal caregiver (*n* = 8). Interviews were digitally recorded with participants’ consent and transcribed verbatim for analysis. For the thematic analysis the data of interviews with 17 participants were used, due to technical issues with one recording and the declining to participate in the interview by two participants.

### Data analysis

Descriptive statistics were used to analyze recruitment, dropout rate, sociodemographic and clinical characteristics of participants and percentage of missing data. After consent, participants were characterized using data on primary tumor, emetogenicity of the chemotherapy, nutritional status and comorbidities from their patient record. It was recorded whether or not outcome measurements were completed. However, no quantitative statistical analyses with the data of the outcome measurements were performed because this study primarily focused on feasibility. Furthermore, sample size calculations were performed before the start of the pilot study and showed that 164 participants were needed to detect a clinically relevant difference in effect of a home delivered meal service on quality of life. The prospective RCT will provide in these numbers and preliminary analysis is not allowed according to the study protocol (ClinicalTrials.gov NCT03382171).

The interview transcripts were analyzed thematically. First, transcripts were coded using the general themes of the topic list. The codes were compared and discussed to reach consensus and to develop a code tree. Thereafter, two researchers discussed and clustered the main categories to identify the main themes (NL, VIJ). Inter-rater reliability was ensured by two researchers coding independently and discussing the interviews and the evolving code tree during the stage of open and axial coding using the software Atlas.ti version 8.1 [31]. Results of the qualitative analysis were discussed within the wider research group for further interpretation and discussion, and here also potential conflicts in coding and analyzing could be resolved [31].

## Results

### Recruitment and dropouts

**Fig. 2:**
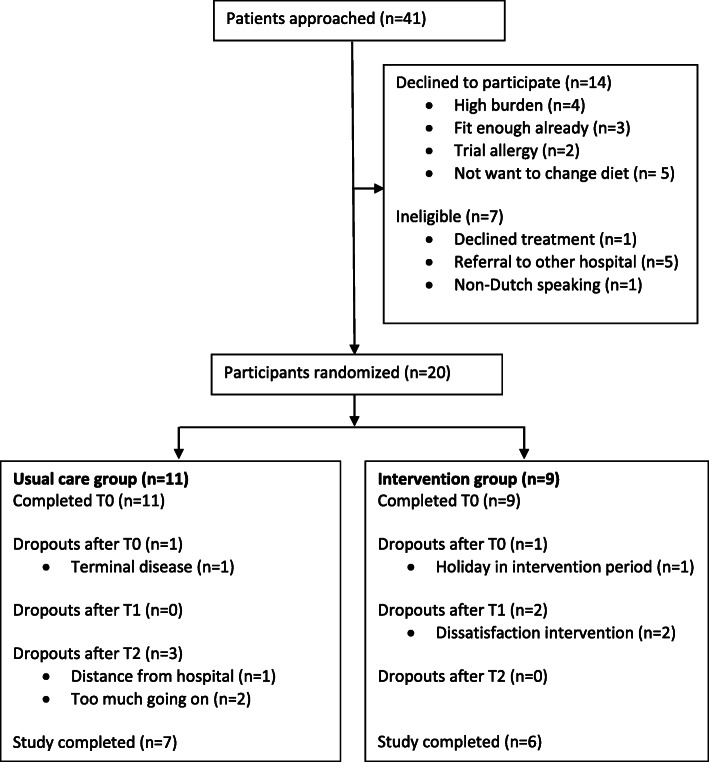
Flowchart of 7 months recruitment in the pilot study

Over a recruitment period of 7 months (November 2017 to June 2018), 41 patients were approached of whom 20 (49%) were included in the study (Fig. 2). Of these 41 patients, 14 declined to participate and 7 were ineligible. Most common reasons for refusal were that some patients considered the burden of participation too high (*n* = 4) and others did not want to change their diet (*n* = 5). The most apparent reason for ineligibility was referral to another hospital for treatment (n = 5).

**Fig. 3:**
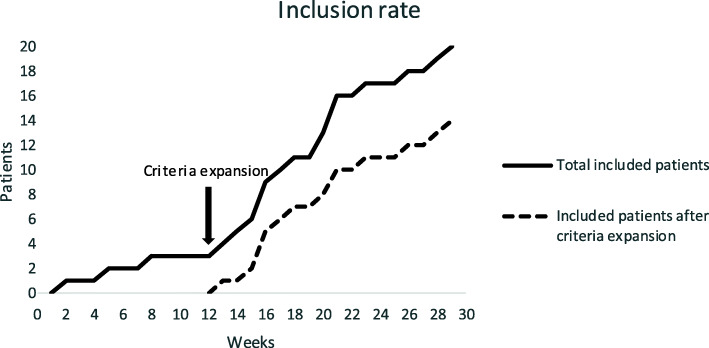
Number of total participants included per week of recruitment and number of included participants after expansion of the in- and exclusion criteria

On average, 6 patients were approached per month (Fig. 3). The inclusion rate was low in the first 3 months (only three patients recruited). This made us decide to expand the inclusion criteria as follows: instead of only recruiting patients with advanced colorectal or gynecological malignancies receiving chemotherapy following a 3-weekly schedule, also curative patients with all cancer types receiving chemotherapy following at least a 2-weekly schedule were eligible because of the similar course of symptoms. Furthermore, since the intervention was not suited for patients with swallowing or passage problems as in head and neck cancer patients, this was added as an extra exclusion criterium. After approval of the ethics committee, these changes were implemented and the inclusion rate increased from three participants in the first 3 months to 17 participants in the next 4 months.

After randomization, 11 participants were allocated to the usual care group and 9 participants to the intervention group. The mean age in the usual care group was 59 years (36% male) compared to a mean age of 63 years in the intervention group (22% male) (Table 1). Overall, 13 participants (65%) completed the follow-up of the study. In the usual care group, seven participants (64%) completed all four time points, one participant dropped out after T0, no participants dropped out after T1 and 3 participants dropped out after T2. In the intervention group, six participants (67%) completed all four time points, one participant dropped out after T0, 2 after T1 and none after T2. Main reasons for dropouts were dissatisfaction with the intervention (*n* = 2), too much going on (n = 2) and a diagnosis of terminal disease (*n* = 1) (Fig. 2).

**Table 1:** Baseline characteristics of the 20 participants included in the pilot study

Baseline characteristics		Usual care group (***n*** = 11)	Intervention group (***n*** = 9)
Gender, n (%)	Male	4 (36)	2 (22)
Age, years, mean ± SD		59 ± 14	63 ± 10
MUST score, n (%)	0	9 (82)	7 (78)
	1	1 (9)	0 (0)
	≥2	1 (9)	2 (22)
Primary tumour, n (%)	Gastrointestinal	5 (46)	3 (33)
	Breast cancer	2 (18)	2 (22)
	Gynecological	4 (36)	4 (44)
Metastases, n (%)	Yes	7 (64)	5 (56)
	No	4 (36)	4 (44)
Emetogenicity chemotherapy, n (%)	High	1 (9)	0 (0)
	Moderate	5 (46)	7 (78)
	Low	5 (46)	2 (22)
Chemotherapy schedule, n (%)	Two weekly	1 (9)	0 (0)
	Three weekly	10 (91)	9 (100)
Comorbidities, n (%)	None	5 (46)	3 (33)
	Diabetes	2 (18)	0 (0)
	Hypertension	2 (18)	1 (11)
	Cardiovascular	1 (9)	0 (0)
	Other	4 (36)	2 (22)

*MUST* malnutrition universal screening tool, *MUST* 0: low risk of malnutrition, *MUST* 1 medium risk of malnutrition, *MUST ≥2* high risk of malnutrition

### Feasibility of study procedures

All participants who completed the follow-up also finished all questionnaires and procedures at T0, T1, T2 and T3 for quality of life, nutritional status and performance score. Hand grip strength (*n* = 8), the SPPB (*n* = 12) and nutritional intake (n = 8) had the highest rate of missing’s at all time points but especially at baseline due to limited time between inclusion and baseline (Table 2). Main reasons were logistic in nature, such as participants who were already connected to their infusion set at the time of the baseline measurement making it impossible to measure hand grip strength at the hand connected to the infusion set and the SPPB. The food diary also had to be filled in two days prior to the start of the chemotherapy which was not always possible due to the short inclusion period. Also, two participants were not willing to visit the hospital earlier to undergo measurements because of the extra burden.

**Table 2:** Number of missing values per study procedure at each time point

	Self-reported	T0 (***n*** = 20)	T1 (***n*** = 18)	T2 (***n*** = 16)	T3 (***n*** = 14)
Quality of life patient (EORTC-QLQ C30)	x	0	0	0	1
Quality of life caregiver (CRA)	x	0	0	0	0
Nutritional status (PG-SGA)		0	0	0	0
Hand grip strength		8	3	3	2
SPPB		12	3	3	3
Karnofsky score		0	0	0	0
Nutritional intake (food diary)	x	8	3	3	2
Symptom- and medication diary^a^	x	NA	NA	2	NA

*NA* not applicable

^a^The symptom and medication diary was only filled in during the intervention period and handed in at T2

### Patient satisfaction

Eighteen out of twenty participants participated in an interview regarding their satisfaction and perceived burden of the participation in this pilot study. Two participants declined to participate in the interview because of the extra burden. In total, the data of interviews with 17 participants (17/20) were used because one interview was not properly recorded due to technical problems.

Most participants expressed that their main reason for participating in the study was to help future patients. One participant even specifically expressed that she participated to help her family and children in case they would get sick in the future. Also, participants felt that the intervention might have positive effects on their health and expressed interest in the topic. For some participants in the intervention group participating was experienced as a positive event because of the nutritional advice they received during the study.

*‘It [participating in the trial] was very useful to me because I got a list with the amount of protein per meal and a guideline about how much protein I needed [during chemotherapy] and that helped me a lot. I used these as guidelines for my meals for weeks afterwards.’*

Of 17 participants, 88% (*n* = 15) graded the level of burden (from 0 to 10) that they experienced by participating in the pilot study. The median level of burden was 2, ranging from 0 to 3. Reasons for this low burden were that participating did not take a lot of extra time or costs because the study procedures could be performed in between their hospital appointments which saved them time and additional parking costs. Several participants said that they would have hesitated or refused to participate if extra visits to the hospital were part of the study considering the high number of regular hospital visits already. Reasons for higher burden were the symptom diary which was considered as too much work and the fact that everything was on paper instead of digitally. Some remarks were made about other cancer patients who were considered more ill or older and therefore, might experience more difficulties in participation.

*‘Of course I do have some complaints but when I see other people in the hospital I think that many people have many more complaints than I do. Well, I really wondered when filling in [the questionnaires] how do people who are really sick do that?’*

Participants expressed their satisfaction with regard to the contact with the study personnel during the study. Participants were also positive about the study personnel giving them instructions in order to enable them to fill in the questionnaires. However, some participants mentioned that participating in such a study did imply contact with many different staff members which was seen as a negative aspect.

### Experiences with study procedures

Participants experienced little problems with completing the quality of life questionnaires. The frequency and time needed for filling in were not experienced as burdensome by most participants and the questionnaire was not confronting. The food diary was also no problem, although some help from their caregiver or the study personnel was sometimes needed. Two participants made remarks about whether the diary was representative for their normal eating habits.

Some participants experienced completing the symptom diary as burdensome, especially because it had to be filled in daily. Others found it a useful tool tracking their symptoms for themselves. One participant even brought the diary to the appointment with the oncologist and used it to express the symptom burden.

*‘So I thought I will bring it [symptom diary] with me so I can show the doctor exactly on which day I had complaints. This way, the doctor could respond with medication so I would have less complaints.’*

The caregivers in this pilot study also completed a questionnaire. Most caregivers experienced little burden, although one caregiver indicated that the Caregiver Reaction Assessment was confronting because it made the caregiver reflect on the course of the disease.

## Discussion

The aim of this study was to assess the feasibility of a pilot RCT on a home delivered meal service in advanced cancer patients undergoing chemotherapy, as compared to usual care. We found an inclusion rate of 49% and an additional dropout rate of 35%, with hand grip strength, SPPB and nutritional intake having the highest number of missing data at all time points, due to logistical reasons. Overall, participants were satisfied with their participation and graded the burden of participating with a median of 2 (0–3).

A delayed recruitment is a well-known phenomenon and is reported in other RCTs. Common causes reported in other RCTs are i) lower numbers of eligible patients than expected, ii) recruiting clinicians forget to invite patients to participate or iii) patients have strong treatment preferences and dislike randomization [32–34]. In addition, there are specific aspects in cancer trials such as reluctancy to participate because of the possible toxic effects of chemotherapy, the belief that a clinical trial is not appropriate for serious diseases and logistical concerns about protocols being too complex [35, 36]. In the present study, we decided to expand our inclusion criteria following disappointing initial inclusion numbers. This expansion resulted in a more heterogenous population and, as expected, an increased inclusion rate. Also, we felt that these expanded criteria better reflect clinical practice and might increase the generalizability of the results. Similarly, a recent pilot focusing on providing meals to older adults after discharge also experienced slow recruitment. These investigators chose not to adapt the inclusion criteria, which resulted in a recruitment period of more than a year to include 24 patients [37]. Besides expanding the inclusion criteria other reported strategies to improve recruitment are i) using a telephone reminder to contact non-responders, ii) keeping the recruiting staff motivated and iii) expanding the recruitment to other hospitals [37, 38]. It could also be useful to estimate the number of eligible patients in advance based on the in- and exclusion criteria. Instructing the clinical nurse specialist to provide the information leaflet as soon as possible is important as well as keeping them motivated for recruitment. Possible strategies to keep recruiting staff motivated could be to i) send them newsletters about the study, ii) speak with them face-to-face about eligible patients, iii) involve them in the protocol writing in order to make them part of the study or iii) share positive responses of participants with them. The process of including other centers takes time and can complicate logistic processes so it is recommended to start this process as soon as a delayed recruitment is noticed and to consider whether the logistics are feasible. However, our findings show that, when approached, patients are willing to participate (50%). As an important reason to participate in the present study, participants expressed they found it important to contribute to science and to contribute to future care for other patients.

In the present study, some patients wanted to start chemotherapy first and dependent on the impact decide whether or not they would be able to participate. In addition, some patients felt that nutritional intervention immediately at the start of the chemotherapy was unnecessary because they did not experience problems with eating and were in good shape. Symptom burden and nutritional outcomes often deteriorate over the course of chemotherapy [39–41]. Therefore, to improve patient inclusion in future studies it seems appropriate to offer patients a nutritional intervention when nutritional impact symptoms occur although implications for internal validity should be kept in mind.

During the study period, 7/20 participants dropped out at one of the four time points. In addition, the highest number of missing values were reported at baseline due to limited time between inclusion and baseline. This is a logistic barrier that is difficult to prevent because it depends on the timing of consent, the often immediate start of the chemotherapy and the treatment plan of the oncologist. A possible strategy could be to maintain close contact with the clinical nurse specialist about the planning of patients. The short time between diagnosis and start of treatment resulting in a short time period available for baseline data collection is also mentioned in another feasibility study [38].

In general, study procedures were not experienced as burdensome and planning of these procedures in line with fixed hospital appointments contributed to this low burden. Several participants indicated that because they were in good shape, the burden of the procedures was experienced as low which was also one of their considerations to participate. On the contrary, higher burden of participation was expressed in regard to the symptom diary and the fact that it had to be filled in every day for 3 weeks. Coolbrandt et al. described that one third of patients found filling in a symptom diary every day too burdensome [42]. Despite the experienced high burden, we received complete symptom diaries of 90% of the participants indicating high compliance. Such diaries have shown to support patients in symptom management which reduces symptom burden [30, 42, 43]. Our participants experienced the diary as a useful tool to track symptoms and communicating this to the oncologist. Therefore, it is important to emphasize the advantage of keeping the diary to the patient and the opportunity it brings in the communication with their physicians. If available, it could also help if the diary could be filled in digitally. Findings on the food diary were that some participants doubted if their contribution was representative and some participants did not fully understand how extensively the food diary needed to be filled in. We therefore recommend to carefully instruct participants on how to fill in the questionnaires and to evaluate this afterwards to prevent misunderstandings for further measurements. Furthermore, the benefits of these questionnaires or diaries for the patient should be part of this instruction to motivate participants and increase compliance.

The present study contributes to a better understanding of patients’ experiences when participating in a nutritional intervention trial during chemotherapy. Furthermore, it describes the challenges as well as possible solutions that arise in the recruitment of patients and data collection during such a trial (Table 3). Another strength is that we performed qualitative interviews with the participants that provide significantly more information when compared with a questionnaire on paper. A limitation of this study was that we could not blind participants nor researchers. Conducting double-blind placebo-controlled trials in nutritional intervention studies is challenging but it does minimize the risk of bias [17, 44]. However, interventions on nutrient supplementation are easier to conduct in a blinded and placebo-controlled manner than whole menu or dietary advice interventions as in our study [17, 44]. Nevertheless, the study participants can be coded in such a way that the researcher analyzing the data is unaware of the group allocation.

**Table 3:** Summary of possible strategies that might improve feasibility in terms of recruitment, missing values and patient satisfaction

Recommendations	
Recruitment and inclusion	- Expand inclusion criteria to increase the number of eligible patients and improve generalizability. - Include more recruitment centers to increase the number of eligible patients as soon as a delayed recruitment is noticed. - Inform eligible patients about the possible course of nutritional symptoms during chemotherapy when asking them to participate. - Inform eligible patients that the burden of participation will be kept as low as possible (no additional hospital visits, non-invasive procedures etc.). - Keep recruiting staff motivated by sending them newsletters about the study, speak with them face-to-face about eligible patients, involve them in the protocol writing or share positive responses of participants with them. - Estimate the number of eligible patients in advance based on the in- and exclusion criteria.
Missing values	- Maintain close contact with the clinical nurse specialist about the planning of patients to prevent missing values at baseline. - Pay extra attention to instructing participants on how to fill in questionnaires and diaries and evaluate afterwards. - Emphasize the benefits of questionnaires and diaries in the communication with their practitioners to create motivation and increase compliance.
Patient satisfaction	- Combine study appointments with existing hospital appointments to prevent extra hospital visits. - Minimize the number of different staff members having contact with the participants. - Keep an overview of the studies that eligible patients are being asked to participate in to prevent that patients are overwhelmed with studies.

## Conclusions

In conclusion, it is feasible to conduct a RCT on a home delivered meal service in advanced cancer patients during chemotherapy. However, it is important to be critical on recruitment goals and to intervene in time when inclusion is lower than expected. Possible strategies are to include additional recruiting centers and to expand in- and exclusion criteria. A logistic barrier was encountered due to the short time frame between consent and baseline procedures which exemplifies the importance of maintaining close contact with the recruiting personnel. Participants experienced low burden with filling in the questionnaires and performing the study procedures. To increase compliance, it is important to carefully instruct participants on how to fill in questionnaires and diaries and to emphasize to use these in the communication with their practitioners. Overall, this study provides guidance for future studies focusing on nutritional interventions in cancer patients receiving chemotherapy.

## Supplementary Information

**Additional file 1.** Semi-structured topic list.

## Data Availability

The datasets used and/or analyzed during the current study are available from the corresponding author on reasonable request.

## Abbreviations

RCT: Randomized controlled trial
SPPB: Short physical performance battery
MDRD-GFR: Modification of diet in renal disease - glomerular filtration rate
FfC: FoodforCare
Kcal: Kilocalorie
CNS: Clinical nurse specialist
EORTC-QLQ C30: European Organization for Research and Treatment for Cancer Quality of Life Questionnaire
CRA: Caregiver reaction assessment
PG-SGA: Patient generated- subjective global assessment
KPS: Karnofsky performance status
