# Supplementary material for: Lessons learned from a randomized controlled trial on a home delivered meal service in advanced cancer patients undergoing chemotherapy: a pilot study
Source: BMC Nutr. 2021 Feb 16;7:4. doi: 10.1186/s40795-021-00407-5 (PMC7885490; doi:10.1186/s40795-021-00407-5)
Supplement: Supplementary file 1 — Additional file 1. Semi-structured topic list. [file 40795_2021_407_MOESM1_ESM.docx]

**Additional file A.** Semi-structured topic list

Experiences with the pilot study

1. **What do you think about participating in this study?**
2. **How did you experience filling in the questionnaires?**

*(think of emotional burden, time burden, physical burden, frequency)*

1. **How did you experience the physical measurements performed during the study?**

*(think of emotional burden, time burden, physical burden, frequency)*

1. **How did you experience filling in the food diary?**

*(think of emotional burden, time burden, physical burden, frequency)*

1. **How would you grade the burden of your participation in this study on a scale from 1 to 10?**

|  | 0 | 1 | 2 | 3 | 4 | 5 | 6 | 7 | 8 | 9 | 10 |  |
| --- | --- | --- | --- | --- | --- | --- | --- | --- | --- | --- | --- | --- |
| No burden  at all | ☐ | ☐ | ☐ | ☐ | ☐ | ☐ | ☐ | ☐ | ☐ | ☐ | ☐ | Worst possible burden |

If partner is present:

1. **What do you think of your partner participating in this study?**
2. **How did you experience the burden of the participation for your partner?**

*(think of emotional burden, time burden, physical burden, frequency)*

1. **How did you experience the burden of the participation for yourself?**

*(think of emotional burden, time burden, physical burden, frequency)*
